# The Inhibitory Effects of Cyclodepsipeptides from the Entomopathogenic Fungus *Beauveria bassiana* on Myofibroblast Differentiation in A549 Alveolar Epithelial Cells

**DOI:** 10.3390/molecules23102568

**Published:** 2018-10-08

**Authors:** Yong Joo Park, Seoung Rak Lee, Dong Min Kim, Jae Sik Yu, Christine Beemelmanns, Kyu Hyuck Chung, Ki Hyun Kim

**Affiliations:** 1 School of Pharmacy, Sungkyunkwan University, Suwon 16419, Korea; pyj084@msn.com (Y.J.P.); davidseoungrak@gmail.com (S.R.L.); kdm9947@gmail.com (D.M.K.); jsyu@bu.edu (J.S.Y.); khchung@skku.edu (K.H.C.); 2 Leibniz Institute for Natural Product Research and Infection Biology-Hans-Knöll-Institute, Beutenbergstraße 11a, 07745 Jena, Germany; Christine.beemelmanns@hki-jena.de

**Keywords:** pulmonary fibrosis, *Beauveria bassiana*, cyclodepsipeptides, myofibroblast differentiation, extracellular matrix (ECM)

## Abstract

Pulmonary fibrosis (PF) is a chronic and fatal lung disease with few treatment options. Although the pathogenesis of PF is not clear, a chronic inflammatory response to continuous damage is considered the cause of pulmonary fibrosis. PF is characterized by excessive accumulation of extracellular matrix (ECM), therefore, inhibition of myofibroblast differentiation is a good therapeutic target for PF. As part of our continuing endeavor to explore biologically active metabolites from insect-associated microbes, we found that the MeOH extract of the culture broth from the entomopathogenic fungus *Beauveria bassiana* inhibited collagen induction and *E*-cadherin down-regulation. In order to identify active compounds, we carried out chemical analysis of the MeOH extract with the assistance of LC/MS-guided isolation approach, which led to the successful identification of four cyclodepsipeptides **1**–**4**. Among the isolates, compound **2** showed inhibitory effects on myofibroblast differentiation induced by TGF-β1. Compound **2** inhibited induction of α-SMA and *N*-cadherin, which are myofibroblast markers, and blocked the accumulation of ECM proteins such as collagen and fibronectin. Overall these findings demonstrate that compound **2** can be used to attenuate pulmonary fibrosis by targeting myo- fibroblast differentiation.

## 1. Introduction

Pulmonary fibrosis (PF) is a chronic lung disease, and chronic inflammation in response to continuous damage is considered to be a major cause [1]. The pathogenesis of PF is not clear. However, it is believed that myofibroblasts play a key role in the development of PF. Myofibroblasts are formed from multiple origins, including resident fibroblasts, circulating bone marrow-derived progenitors, and alveolar epithelial cells (AECs), through a process termed “epithelial mesenchymal transition” (EMT) [1,2]. When there is injury to the lung, inflammation and oxidative stress increase, which accelerates the process of differentiation into the myofibroblast phenotype [2]. Myofibroblasts are characterized by high expression of α-smooth muscle actin (α-SMA; gene *ACTA*) and collagen, leading to extracellular matrix (ECM) deposition [3,4]. 

PF is characterized by excessive accumulation of ECM proteins. ECM plays an important role in wound healing and organ homeostasis by regulating cell morphology, cell-cell interaction, and cellular differentiation and migration [5]. Therefore, ECM homeostasis is controlled by various enzymes such as matrix metalloproteinases (MMPs) and tissue inhibitor of metalloproteinase-1 (TIMP-1) [6]. However, chronic damage provokes excessive ECM production and can result in life-threatening conditions such as fibrotic diseases and cancers [5].

Entomopathogenic fungi are the most common disease-causing microbes in insects and have evolved to make biologically active small molecules that suppress other pathogens, such as bacteria, virus, and other pathogenic fungi [7]. Recent advances in the genome biology of entomopathogenic fungi suggest that these fungi are a promising natural source for exploring structurally and/or biologically unique compounds [8]. In previous studies on biologically active metabolites from entomopathogenic fungi, destruxin A (cyclic hexadepsipeptide) isolated from *Metarhizium anisopliae* was found to exhibit insecticidal, cytotoxic, and antibacterial effects [9]. In addition, the red 1,4-bibenzoquinone derivative, oosporein, identified from *Beauveria bassiana,* showed antibiotic, antifungal, antiviral, and insecticidal activities [10].

As part of our continuing endeavor to explore biologically active metabolites from insect-associated microbes [11,12,13,14], we found that the MeOH extract of the culture broth of the entomopathogenic fungus *B. bassiana* blocked collagen expression induced by TGF-β1. It also increased *E*-cadherin expression that had been down-regulated by TGF-β1. In addition, comprehensive LC/MS analysis of the MeOH extract, including a built-in UV spectra library, revealed the existence of peptidic-type metabolites. Chemical analysis of the MeOH extract with the assistance of an LC/MS-guided isolation approach led to the successful identification of four cyclodepsipeptides (**1**–**4**). To assess the effects of isolated compounds on myofibroblast differentiation, we analyzed the expression of protein and mRNA associated with myofibroblast differentiation on compounds treated with TGF-β1. Also, we measured the mRNA expression of myofibroblast-associated genes to elucidate the pathway of anti-fibrotic effects for further mechanisms. In this paper, we report the chemical identification of four cyclodepsipeptides **1**–**4**, their anti-fibrotic effects against A549 human lung alveolar epithelial cells, and the underlying mechanism of action.

## 2. Results and Discussions

### 2.1. The Inhibitory Effects of MeOH Extract of B. bassiana on Myofibroblast Differentiation

**Figure 1:**
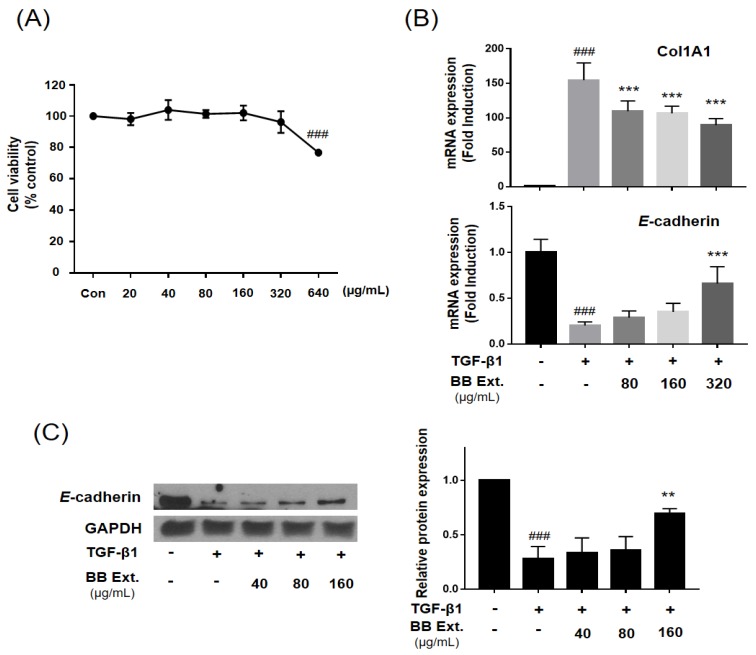
MeOH extract of the culture broth of the entomopathogenic fungus *B. bassiana* (BB) inhibited TGF-β1-induced myofibroblast differentiation in alveolar epithelial A549 cells. A549 cells were treated with TGF-β1 (2 ng/mL) and MeOH extract for 48 h. (**A**) Cytotoxicity of MeOH extract in A549 cells was measured by WST-1 assay at 48 h. (**B**) Expression of collagen (Col1A1) and *E*-cadherin was analyzed by qPCR-analysis. (**C**) The protein expression of *E*-cadherin was analyzed by western blot assay. All the assays were performed in triplicate, and the data are shown as the mean value ± S.D. Values that are significantly different from the control are indicated by ^###^ *p* < 0.001, significantly different from the TGF-β1-treatment group, ** *p* < 0.01, or *** *p* < 0.001.

*B. bassiana* (ST000047, Jena Microbial Research Collection) was cultivated in standing liquid culture (PDB), and combined MeOH extract from the resultant mycelium and supernatant was obtained. In our screening test, cytotoxicity of the MeOH extract was measured after 48-h treatment with WST-1 assay (Figure 1A). The MeOH extract showed cytotoxicity in a dose-dependent manner, and the concentration without cytotoxicity (40–320 μg/mL) was selected for testing inhibitory effects on myofibroblast differentiation. The MeOH extract treatment decreased the mRNA expression of collagen (collagen 1A1), a myofibroblast marker, and increased the expression of *E*-cadherin, an epithelial marker (Figure 1B). The MeOH extract also significantly increased protein expression of *E*-cadherin at 160 μg/mL, which had been down-regulated by TGF-β1 (Figure 1C). Epithelial cells are an important source of myofibroblast differentiation in PF via the EMT process, and TGF-β1 is a major growth factor that induces EMT [2]. Adding TGF-β1 to epithelial cells in culture is the most common way to induce EMT, and we used TGF-β1 to induce a myofibroblast-like phenotype [15]. We observed significantly decreased mRNA and protein expression of *E*-cadherin which is considered as a hallmark of EMT after treatment of TGF-β1. As shown in Figure 1, our results indicated that MeOH extract of the culture broth of *B. bassiana* is a possible inhibitor in the process of myofibroblast differentiation.

### 2.2. LC/MS-Based Isolation and Identification of Compounds ***1**–**4***

**Figure 2:**
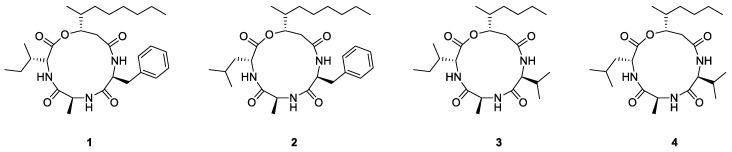
The structures of cyclodepsipeptides **1**–**4** isolated from culture broth of *B. bassiana*.

In order to identify active compounds, the MeOH extract was solvent partitioned with EtOAc to obtain the EtOAc-soluble fraction, which was then subjected to repeated column chromatography and semi-preparative HPLC purification with the assistance of LC/MS-guided isolation approach (see Supplementary Materials). The process resulted in the successful isolation of four cyclodepsipeptides **1**–**4** (Figure 2). Using extensive NMR spectroscopic methods and comparing our results with previously reported spectroscopic values, as well as LC/MS analysis, the the isolates were determined to be beauverolide La (**1**) [16], beauverolide L (**2**) [16], beauveriolide V (**3**) [17], and beauveriolide VI (**4**) [17]. Cyclodepsipeptides encompass a wide variety of cyclic peptides from natural origin and are identified by the inclusion of at least one ester linkage. Cyclodepsipeptides have been recognized to have a variety of biological activities according to previously reported literature, including anti-inflammatory, antimicrobial, antiproliferative, and immunosuppressant effects [18,19,20,21]. To the extent of our knowledge, anti-fibrotic effects of cyclodepsipeptides by targeting anti-EMT properties have to date not been investigated.

### 2.3. The Inhibitory Effects of Cyclodepsipeptides ***1**–**4*** on Myofibroblast Differentiation

**Figure 3:**
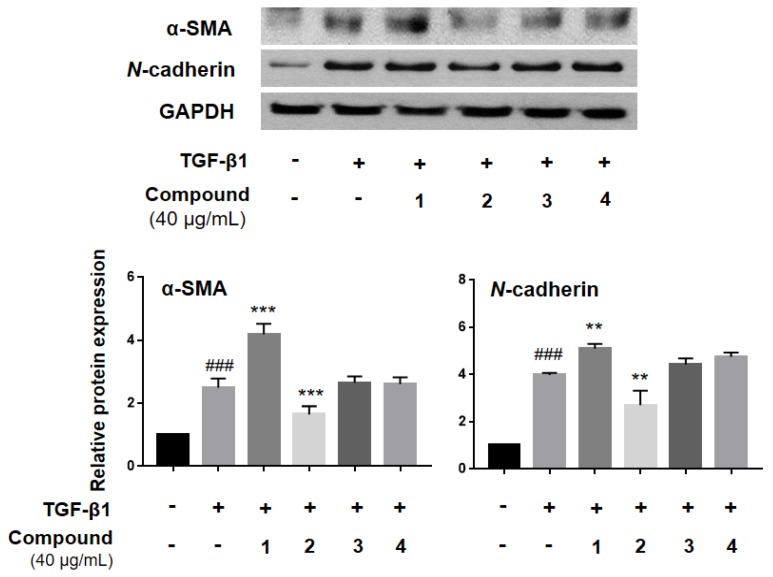
Compound **2** inhibited TGF-β1-induced myofibroblast differentiation in alveolar epithelial A549 cells. A549 cells were treated with TGF-β1 (2 ng/mL) and isolate compounds (40 µg/mL) for 48h. The protein expression of α-SMA and *N*-cadherin was analyzed by western blot. All the assays were performed in triplicate, and the data are shown as the mean value ± S.D. Values that are significantly different from the control are indicated by ^###^ *p* < 0.001, and values that are significantly different from the TGF-β1-treated group are indicated by ** *p* < 0.01, and *** *p* < 0.001.

Since all isolated cyclodepsipeptides **1**–**4** were purified from an active MeOH extract that inhibited myofibroblast differentiation, these compounds were then tested individually for their ability to inhibit the expression of α-SMA and *N*-cadherin, which are myofibroblast differentiation markers (Figure 3). This expression was induced by TGF-β1 treatment, and compound **2** was found to significantly inhibit α-SMA and *N*-cadherin expression, compared to the TGF-β1-treated group (Figure 3).

### 2.4. The Inhibitory Effects of Compound ***2*** on Myofibroblast Differentiation 

**Figure 4:**
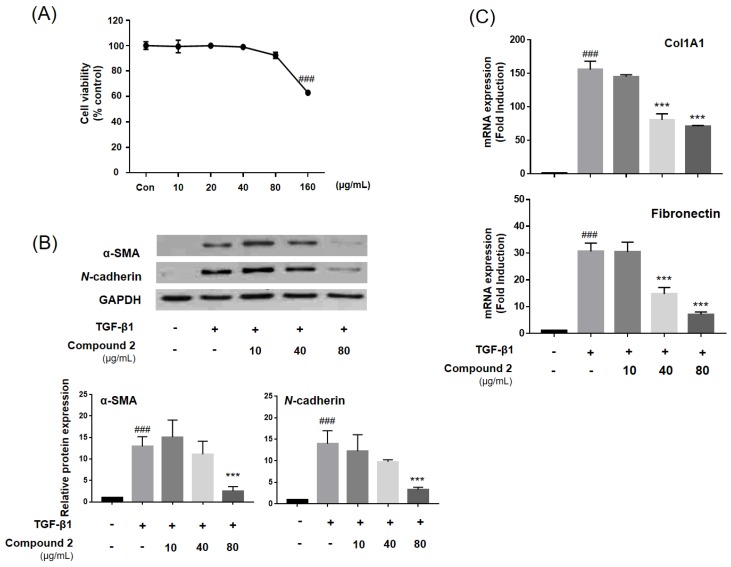
Compound **2** ameliorate TGF-β1-induced myofibroblast differentiation in alveolar epithelial A549 cells. A549 cells were treated with TGF-β1 (2 ng/mL) and compound **2** for 48 h. (**A**) Cytotoxicity of compound **2** in A549 cells was measured by WST-1 assay at 48h. Compound **2** was treated to A549 cells. (**B**) The protein expression of α-SMA and *N*-cadherin was analyzed by western blot. (**C**) Expression of collagen (Col1A1) and fibronectin was analyzed by qPCR-analysis. All the assays were performed in triplicate, and the data are shown as the mean values ± S.D. Values that are significantly different from the control are indicated by **^###^** *p* < 0.001, and values that are significantly different from the TGF-β1-treated group are indicated by *** *p* < 0.001.

The cytotoxicity of compound **2** (0–160 μg/mL) was measured using a WST-1 assay after 48-h treatment (Figure 4A). The concentration without cytotoxicity (0–80 μg/mL) was selected, and A549 human lung alveolar epithelial cells were treated with TGF-β1. The protein and mRNA expression associated with the myofibroblast differentiation was measured. Compound **2** significantly inhibited α-SMA and *N*-cadherin protein expression, which had been induced by TGF-β1 treatment (Figure 4B). Moreover, following analysis by mRNA expression, compound **2** inhibited accumulation of ECM proteins such as type I collagen and fibronectin (Figure 4C). Myofibroblast cells are important in fibrosis development and increase ECM proteins accumulation [22]. Collagen and fibronectin were observed to be increased in myofibroblasts cells, with a-SMA and *N*-cadherin being myofibroblast markers [23]. During the EMT, epithelial cells differentiated to the myofibroblast phenotype, ECM composition and structure were modified, and ECM components such as collagen, laminins, proteoglycans, and fibronectin accumulated [24,25]. Therefore, inhibition of myofibroblast differentiation by targeting the EMT is a good strategy in PF treatment. 

**Figure 5:**
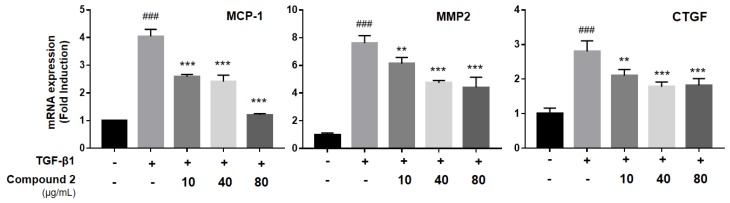
Compound **2** inhibited TGF-β1-induced myofibroblast associated genes in alveolar epithelial A549 cells. A549 cells were treated with TGF-β1 (2 ng/mL) and compound **2** for 48 h. Any expression of MCP-1, MMP2 and CTGF was analyzed by qPCR-analysis. All of the assays were performed in triplicate, and the data are shown as the mean values ± S.D. Values that are significantly different from the control are indicated by **^###^** *p* < 0.001, and values that are significantly different from the TGF-β1-treated group are indicated by ** *p* < 0.01, and *** *p* < 0.001.

We also found out that compound **2** inhibited the expression of monocyte chemoattractant protein-1 (MCP-1), connective tissue growth factor (CTGF), and MMP2 (Figure 5). MCP-1, CTGF and MMP2 are all important factors in pulmonary fibrosis development. MCP-1 is a proinflammatory chemokine that recruits monocytes into the inflammation site after induction by oxidative stress, cytokines, or growth factors [26]. It also may induce EMT in epithelial cells by phosphorylation of Erk1/2 and Akt proteins [27,28]. CTGF is important in cell adhesion, migration, and myofibroblast activation. Several studies indicate that blockage of CTGF ameliorates fibrosis through inhibiting EMT [29,30]. MMPs are protease enzymes that are responsible for degrading ECM and are considered important enzymes in ECM homeostasis [6]. MMPs were observed in high levels in diseased or inflamed organs and are highly-correlated with pro-fibrotic mediators [31]. In our study, compound **2** treatment inhibited up-regulation of MMP2 by TGF-β1 treatment. The dissolution of the epithelial junction enhanced cell mobility. In addition, it increased the release of MMPs, especially MMP2 and MMP9, which can degrade the collagens [31]. Because compound **2** inhibited myofibroblast differentiation markers and ECM proteins, our finding demonstrates that compound **2** can inhibit the EMT process by targeting MCP-1, MMP2, and CTGF pathways.

## 3. Materials and Methods 

### 3.1. General Experimental Procedures 

Optical rotations were calculated using a P-1020 polarimeter (Jasco, Easton, MD, USA). IR spectra were acquired on an IFS-66/S FT-IR spectrometer (Bruker, Madison, WI, USA). LC/MS analysis was performed on an Agilent 1200 Series HPLC system (Santa Clara, CA, USA) equipped with a diode array detector and a 6130 Series ESI mass spectrometer using a Kinetex C18 100 Å analytical column (100 mm × 2.1 mm i.d., 5 μm). NMR spectra were recorded on a UNITY INOVA 800 NMR spectrometer (Varian, Palo Alto, CA, USA) operating at 800 MHz (^1^H) and 200 MHz (^13^C), with chemical shifts given in ppm (δ). Preparative high-performance liquid chromatography (HPLC) used a Waters 1525 Binary HPLC pump with a Waters 996 Photodiode Array Detector (Waters Corporation, Milford, CT, USA). Semi-preparative HPLC utilized a Prominence HPLC System with SPD-20A/20AV Series Prominence HPLC UV-Vis Detectors (Shimadzu, Tokyo, Japan). Silica gel 60 (70–230 mesh and 230–400 mesh, Merck, Darmstadt, Germany) and RP-C18 silica gel (Merck, 40–63 μm) were used for column chromatography. Merck precoated silica gel F254 plates and RP-18 F254s plates were used for TLC. Spots were detected on TLC under UV light or by heating after spraying with anisaldehyde-sulfuric acid.

### 3.2. Fermentation Procedures

Media cultures: (1) yeast malt agar (YMA): 2.0 g/L yeast extract, 20.0 g/L malt extract, 20.0 g/L agar; (2) potato dextrose broth (PDB): 26.5 g/L potato extract glucose; (3) potato dextrose agar, (PDA): 26.5 g/L potato extract glucose, 20.0 g/L agar. *B. bassiana* (ST000047, Jena Microbial Research Collection) was cultivated on YMA or PDA plates for a maximum of six weeks at 25 °C and was then subcultured by plating mycelium-containing agar pieces (1 × 1 cm) onto fresh YMA or PDA. For liquid cultivation, PDB was inoculated using mycelium-covered agar plugs (1 × 1 cm). 

### 3.3. Extraction Procedures from Liquid Cultures

Approximately 10 mycelium-containing PDA agar pieces (1 × 1 cm) from a four-week old plate culture were used for inoculation of 500 mL liquid medium. In total, 4 × 400 mL culture were incubated at 25 °C for two weeks under static conditions. Mycelium was collected by centrifugation and the combined supernatant mixed with activated HP-20 resin (5 g/100 mL). After stirring overnight at 4 °C, the HP-20 resin was separated from supernatant by filtration, washed with 20% MeOH, and the extracts eluted using 100% MeOH (2 L). In a second step, the collected cell pellet was extracted separately with 500 mL MeOH overnight at 4 °C and filtered, and the resulting MeOH extract was combined with the 100% MeOH HP-20 resin eluent. The combined extracts were dried completely under reduced pressure.

### 3.4. Isolation of Compounds

The crude MeOH extract (10.9 g) was found to inhibit collagen induction and *E*-cadherin down-regulation. Extensive LC/MS analysis of the MeOH extract in our LC/MS system showed non-polar metabolite peaks exhibiting strong UV absorption at around 200–220 nm, with interesting ion peaks at *m/z* 439 and 515, which suggested the possibility of the presence of peptidic-type metabolites. The MeOH extract was dissolved in distilled water (700 mL) and solvent-partitioned with EtOAc (700 mL × 3) to yield the EtOAc-soluble fraction (1.1 g). The EtOAc-soluble fraction, which contained promising peaks on LC/MS analysis, was loaded onto a silica gel (230–400 mesh) column chromatography and fractionated with a gradient solvent system of CH_2_Cl_2_-MeOH (50:1–1:1, *v/v*) to afford seven subfractions (B1–B7). Subfraction B3 (36.5 mg) was isolated by semi-preparative reversed-phase HPLC (Phenomenex Luna C-18 column, 250 × 10.0 mm, 5 μm) eluted with 73% MeOH/H_2_O (flow rate: 2 mL/min) to obtain compounds **3** (2.6 mg, *t*_R_ = 45.0 min) and **4** (2.7 mg, *t*_R_ = 48.0 min). Subfraction B4 (20.4 mg) was purified to acquire compounds **1** (2.7 mg, *t*_R_ = 41.0 min) and **2** (1.6 mg, *t*_R_ = 44.5 min) by semi-preparative reversed-phase HPLC (Phenomenex Luna C-18 column, 250 × 10.0 mm, 5 μm) with 70% MeOH/H_2_O (flow rate: 2 mL/min). 

### 3.5. Cell Lines

Adenocarcinomic human lung epithelial cells, A549 were obtained from the American Type Culture Collection (ATCC, Manassas, VA, USA). Cells were maintained in DMEM (Sigma, St. Louis, MO, USA) supplemented with 10% of FBS and 1% of penicillin/streptomycin solution and grown in a humidified incubator, 95% air, 5% CO_2_, at 37 °C. 

### 3.6. Cell Viability

Cell viability was tested using the WST-1 assay (Roche, Mannheim, Germany). After chemical incubation, WST-1 reagent was added to each well (1:10 final dilution) and incubated for 1 h at 37 °C. Cell viability was quantified by measuring the absorbance at a wave length of 440 nm using VERSAmax microplate reader (Molecular Devices, Sunnyvale, CA, USA). 

### 3.7. Comparative Quantitative Real-Time PCR (qPCR)

Total RNA from cells was extracted with TRIzol reagent (Life Technologies, Grand Island, NY, USA) following the protocol provided by the manufacturer. RNA concentration was measured using BioDrop Duo (Biodrop, Cambridge, UK). cDNA was synthesized from 2 µg of total RNA using the High-Capacity cDNA Reverse Transcription System (Life Technologies). Comparative quantitative RT-PCR (qPCR) was performed in duplicate for each sample using SYBR^®^ Premix Ex Taq^TM^ (Life Technologies) and CFX CFX96 Real-Time PCR System (Bio-Rad, Hercules, CA, USA). RT-PCRs were performed using the primers listed in Table 1. Expression levels of mRNA were quantified by use of the threshold cycle (Ct) method. Ct values for each gene of in­terest were normalized to GAPDH.

### 3.8. Western Blot Analysis

Anti-alpha-SMA (A2547, Sigma), anti-*E*-cadherin (#3195, Cell signaling technology, Danvers, MA, USA), anti-*N*-cadherin (#18203, Abcam, Cambridge, MA, USA), and anti-GAPDH (sc-1694, Santa Cruz Biotechnology, Dallas, TX, USA) were used in western blot analysis. In general, the cells were washed with PBS twice and lysed with radioimmunoprecipitation assay buffer (786–489; G-Biosciences, St. Louis, MO, USA) and a protease inhibitor cocktail (1 mM PMSF and 1 μg/mL each of aprotinin, leupeptin, and pepstatin A). The cells were then collected by scraping into a 1.5 mL microtube and incubated on ice for 10 min. Cells were subsequently centrifuged at 13,000× *g* for 15 min at 4 °C. The lysates were quantified using the Pierce^TM^ BCA Protein Assay Kit (Pierce, Rockford, IL, USA). Samples were denatured with buffer containing 2% SDS, 6% 2-mercaptoethanol, 40% glycerol, 0.004% bromophenol blue, and 0.06 M Tris–HCl at 90–100 °C for 6 min, then cooled at room temperature for 5 min. Denatured total proteins were loaded in each lane of SDS-polyacrylamide gels and transferred to nitrocellulose membrane. The membrane was blocked with 3% bovine serum albumin (BSA, Sigma) for 1 h at room temperature and incubated with primary antibodies overnight. The blots were incubated with the appropriate secondary antibodies conjugated to horseradish peroxidase for 1 h. Protein bands were developed onto X-ray film (Fujifilm, Tokyo, Japan) after incubation with chemiluminescence substrate, and the densities of each band were normalized to those of GAPDH band.

### 3.9. Statistical Analysis

GraphPad Prism 5 software (GraphPad Software, Inc., La Jolla, CA, USA) and Excel 2016 (Microsoft, Redmond, WA, USA) were used to analyze the data. Differences between groups were assessed by one-way analysis of variance followed by Tukey’s post hoc test or a Student’s *t*-test as appropriate. Error bars represent standard deviation (SD) or standard error of the mean (SEM), as indicated. Statistical significance was accepted at *p* < 0.05. 

## 4. Conclusions

Pulmonary fibrosis is a chronic lung disease with few treatment options. Therefore, it is important that effective therapeutic treatment be developed. Our report provides a chemical analysis of biologically active metabolites from the entomopathogenic fungus *B. bassiana*. Four cyclodepsipeptides, beauverolide La (**1**), beauverolide L (**2**), beauveriolide V (**3**), and beauveriolide VI (**4**), were isolated and structurally characterized from the culture broth of *B. bassiana* using an LC/MS-based isolation method. All of the isolated compounds were evaluated for inhibitory properties of myofibroblast differentiation, and compound **2** showed strong effects on A549 human alveolar epithelial cells. We suggest that compound **2** can be utilized to treat pulmonary fibrosis with anti-EMT properties.

## Figures and Tables

**Table 1:** RT-PCR primer sequences.

Gene	Forward	Reverse	Size (bp)
CoL1A1	GGCAACAGCCGCTTCACCTAC	GCGGGAGGACTTGGTGGTTTT	105
*E*-cadherin	TGCCCAGAAAATGAAAAAGG	GTGTATGTGGCAATGCGTTC	200
Fibronectin	CAGTGGGAGACCTCGAGAAG	TCCCTCGGAACATCAGAAAC	168
MCP-1	GTCACCTGCTGTTATAACTTC	TGCTGCTGGTGATTCTTCTA	79
MMP2	GGAAAGCCAGGATCCATTTT	ATGCCGCCTTTAACTGGAG	103
CTGF	CAAGGGCCTCTTCTGTGACT	ACGTGCACTGGTACTTGCAG	146
GAPDH	AATCCCATCACCATCTTCCA	TGGACTCCACGACGTACTCA	82

## Supplementary Materials

The following are available online. NMR data and LC/MS data of compounds **1**–**4** are available free of charge on the Internet.
