# Supplementary material for: The Inhibitory Effects of Cyclodepsipeptides from the Entomopathogenic Fungus Beauveria bassiana on Myofibroblast Differentiation in A549 Alveolar Epithelial Cells
Source: Molecules. 2018 Oct 8;23(10):2568. doi: 10.3390/molecules23102568 (PMC6222899; doi:10.3390/molecules23102568)

Supporting Information

The Inhibitory Effects of Cyclodepsipeptides from the Entomopathogenic Fungus *Beauveria bassiana* on Myofibroblast Differentiation in A549 Alveolar Epithelial Cells

Yong Joo Park [1,#], Seoung Rak Lee [1,#], Dong Min Kim [1], Jae Sik Yu [1], Christine Beemelmanns [2], Kyu Hyuck Chung [1] and Ki Hyun Kim [1,*]

[1] School of Pharmacy, Sungkyunkwan University, Suwon 16419, Republic of Korea; pyj084@msn.com (Y.J.P.); davidseoungrak@gmail.com (S.R.L); kdm9947@gmail.com (D.M.K.); jsyu@bu.edu (J.S.Y.); khchung@skku.edu (K.H.C.)

[2] Leibniz Institute for Natural Product Research and Infection Biology – Hans-Knöll-Institute, Beutenbergstraße 11a, 07745 Jena, Germany; Christine.beemelmanns@hki-jena.de (C.B.)

[#] These authors contributed equality to this work.

* Corresponding author:

Ki Hyun Kim, Tel: +82-31-290-7700; Fax: +82-31-290-7730; E-mail: khkim83@skku.edu

Supporting Information Contents:

Figure S1. The ^{1}H NMR spectrum of **1** (Pyridine-d_5, 800 MHz)........3

Figure S2. The ^{1}H NMR spectrum of **2** (Pyridine-d_5, 800 MHz)........4

Figure S3. The ^{1}H NMR spectrum of **3** (CD_3OD, 800 MHz)........5

Figure S4. The ^{1}H NMR spectrum of **4** (Pyridine-d_5, 800 MHz) 6

Figure S5. UV chromatogram of LC/MS (detection wavelength was set as 210 nm) of the MeOH extract of the culture broth from the entomopathogenic fungus *Beauveria bassiana* 7

Figure S6. The LC/MS-guided isolation of compounds **1** and **2**........8

Figure S7. The LC/MS-guided isolation of compounds **3** and **4**........9

Figure S1. The ^{1}H NMR spectrum of **1** (Pyridine-d_5, 800 MHz)

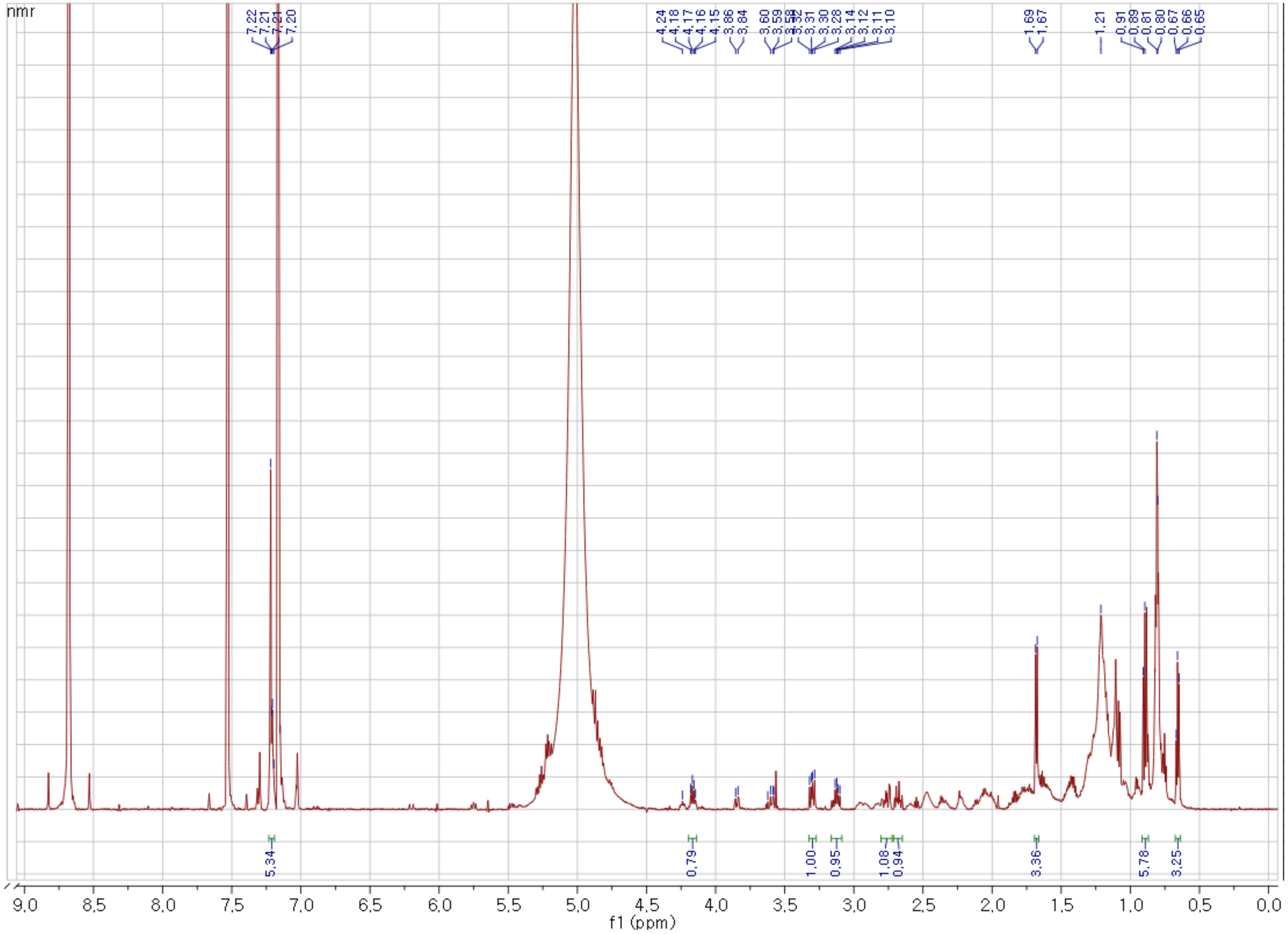

Figure S2. The ^{1}H NMR spectrum of **2** (Pyridine-d_5, 800 MHz)

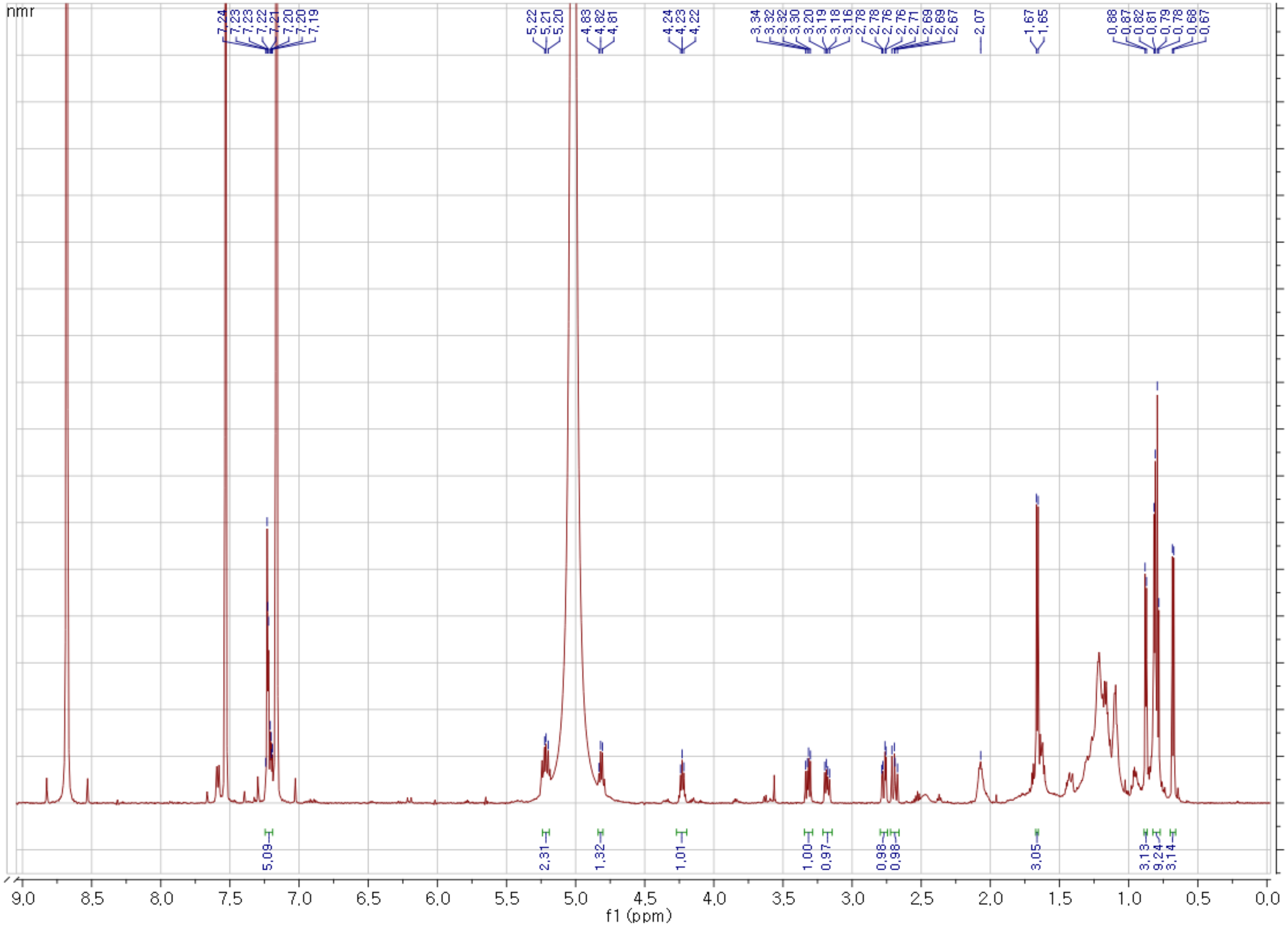

Figure S3. The ^{1}H NMR spectrum of **3** (CD_3OD, 800 MHz)

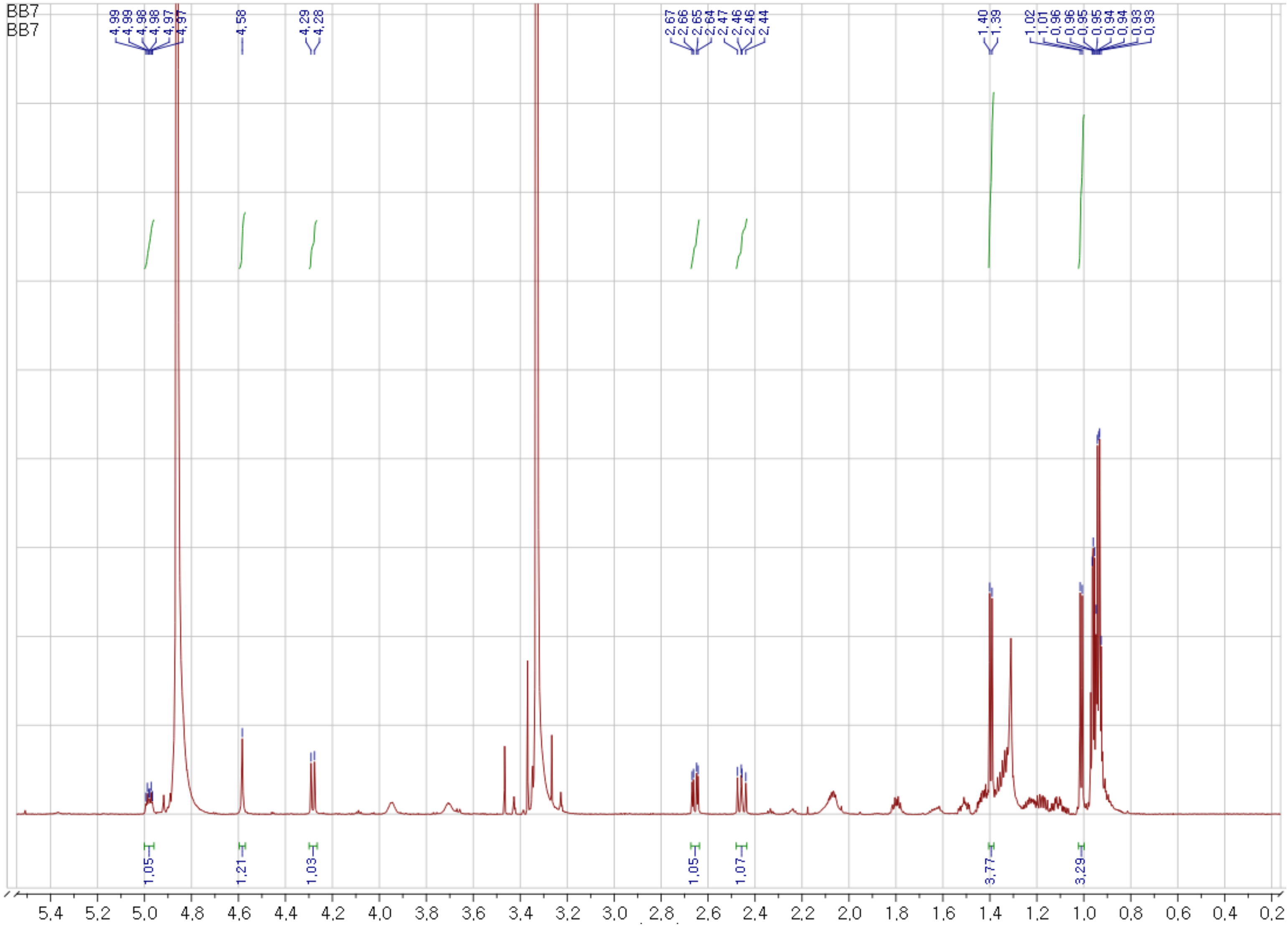

Figure S4. The ^{1}H NMR spectrum of **4** (Pyridine-d_5, 800 MHz)

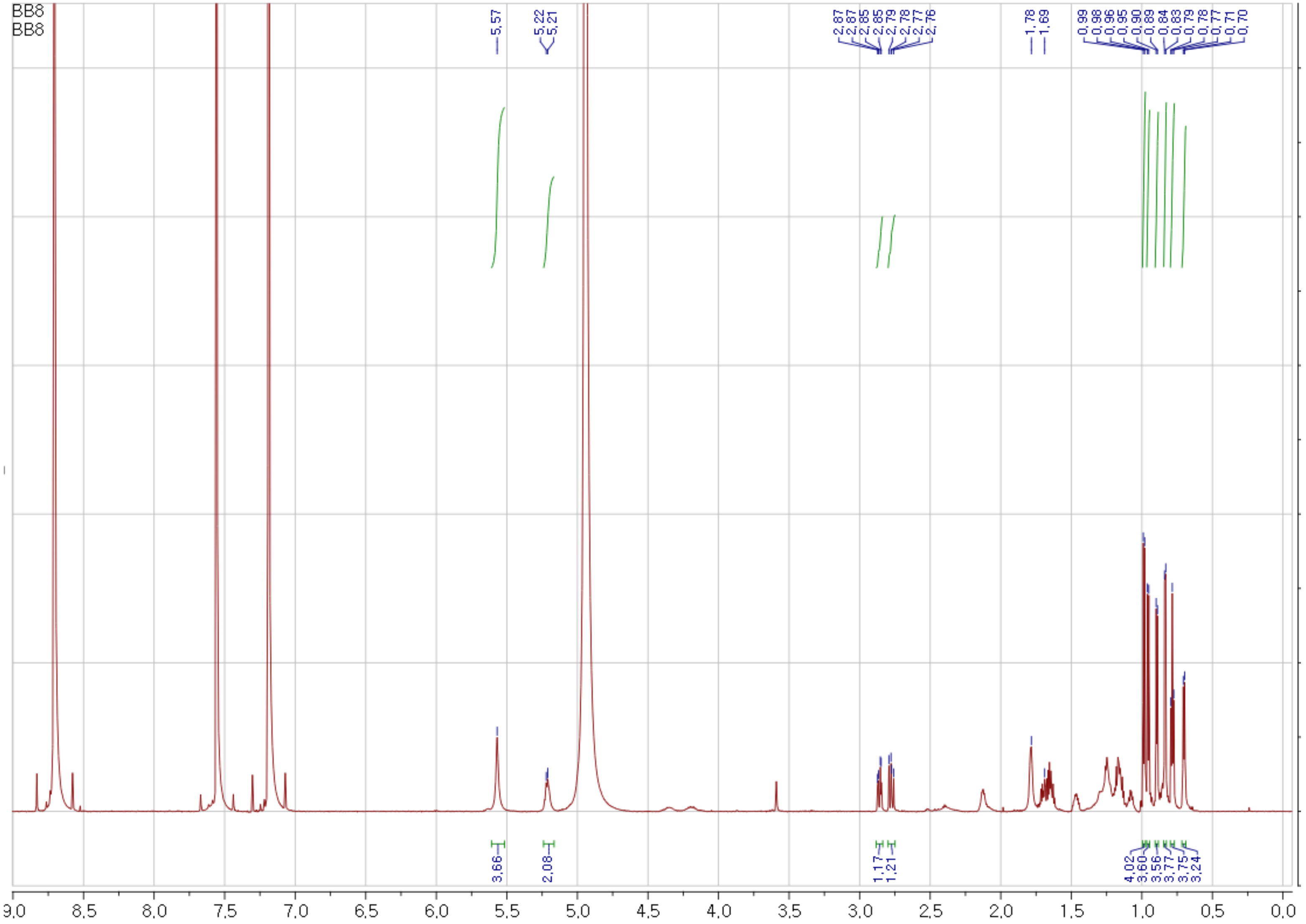

Figure S5. UV chromatogram of LC/MS (detection wavelength was set as 210 nm) of the MeOH extract of the culture broth from the entomopathogenic fungus *Beauveria bassiana*

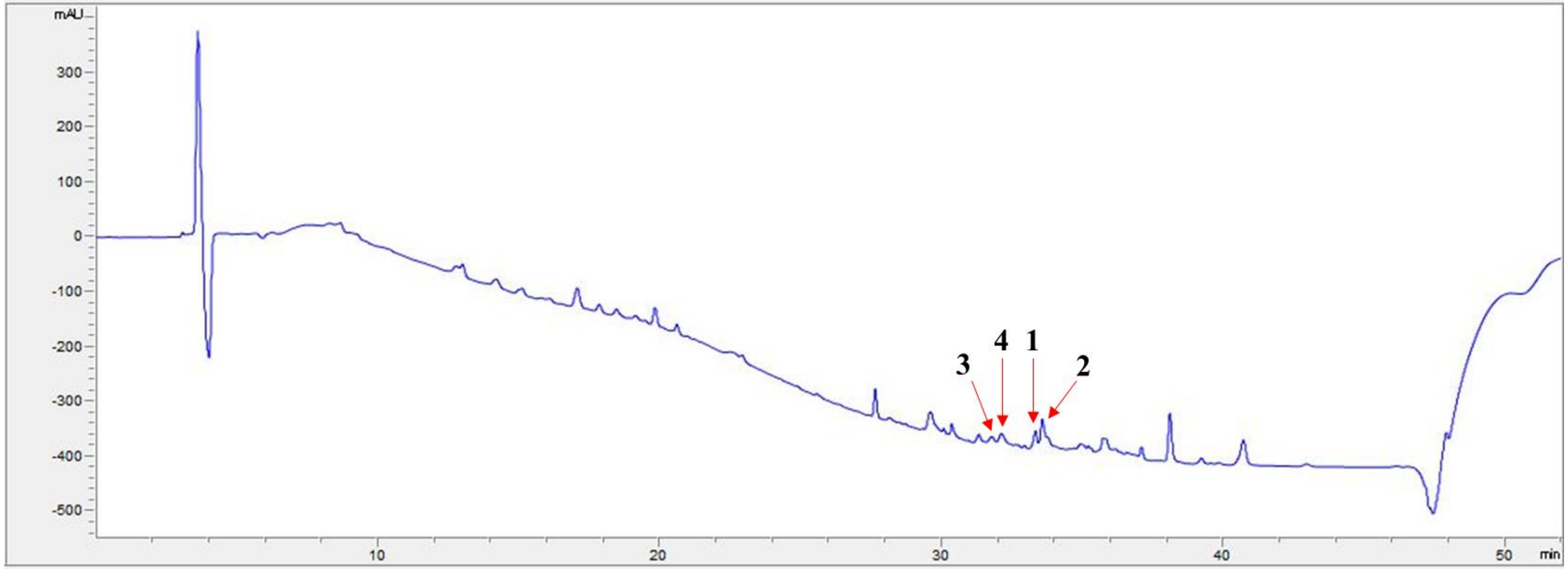

LC/MS analysis:

The MeOH extract of the culture broth from *B. bassiana* was analyzed by LC/MS. Briefly, stock solution of the MeOH extract was prepared by dissolving 1 mg of sample in 1 mL methanol. The solution was further diluted with methanol to provide a solution of 100 μg/mL. The solution was filtered through a 0.45 mm hydrophobic PTFE filter and analyzed by LC/MS (Agilent Technologies, Santa Clara, CA, USA) using a LC-MS Agilent 1200 Series analytical system equipped with a photodiode array (PDA) detector combined with a 6130 Series ESI mass spectrometer. Analysis was performed by injection of 5 μL of the sample using a Phenomenex Luna C18 (4.6 × 100 mm, 3.5 μm) and the full scan in positive and negative ion modes (scan range *m*/*z* 100 to 1000) was applied. The mobile phase consisting of formic acid in H_2O [0.1% (v/v)] (A) and methanol (B) was delivered at a flow rate of 0.3 mL/min by applying the following programmed gradient elution: 10%-100% (B) for 30 min, 100% (B) for 1 min, 100% (B) isocratic for 10 min, and then 10% (B) isocratic for 10 min, to perform post-run reconditioning of the column.

Figure S6. The LC/MS-guided isolation of compounds **1** and **2**

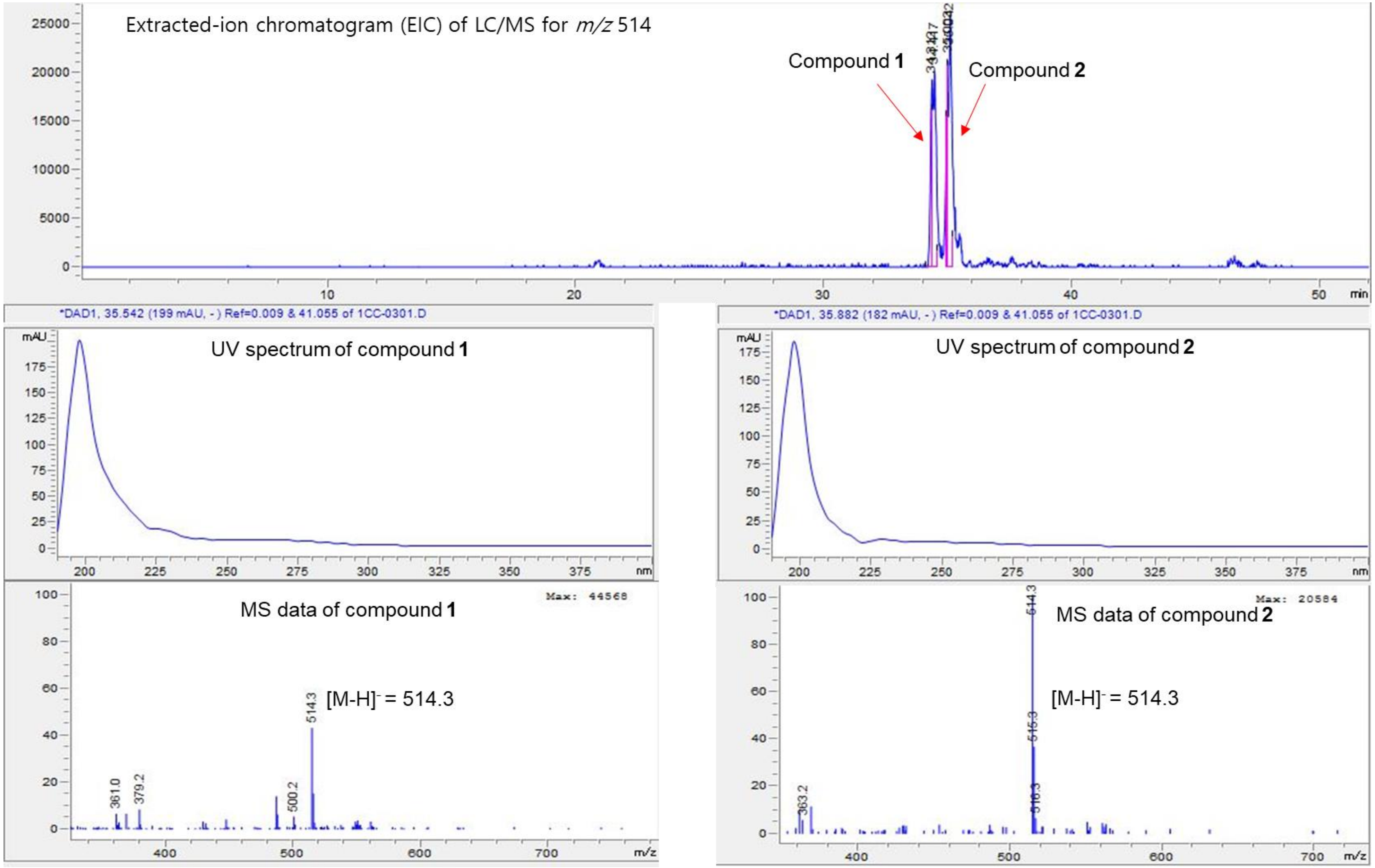

Figure S7. The LC/MS-guided isolation of compounds **3** and **4**

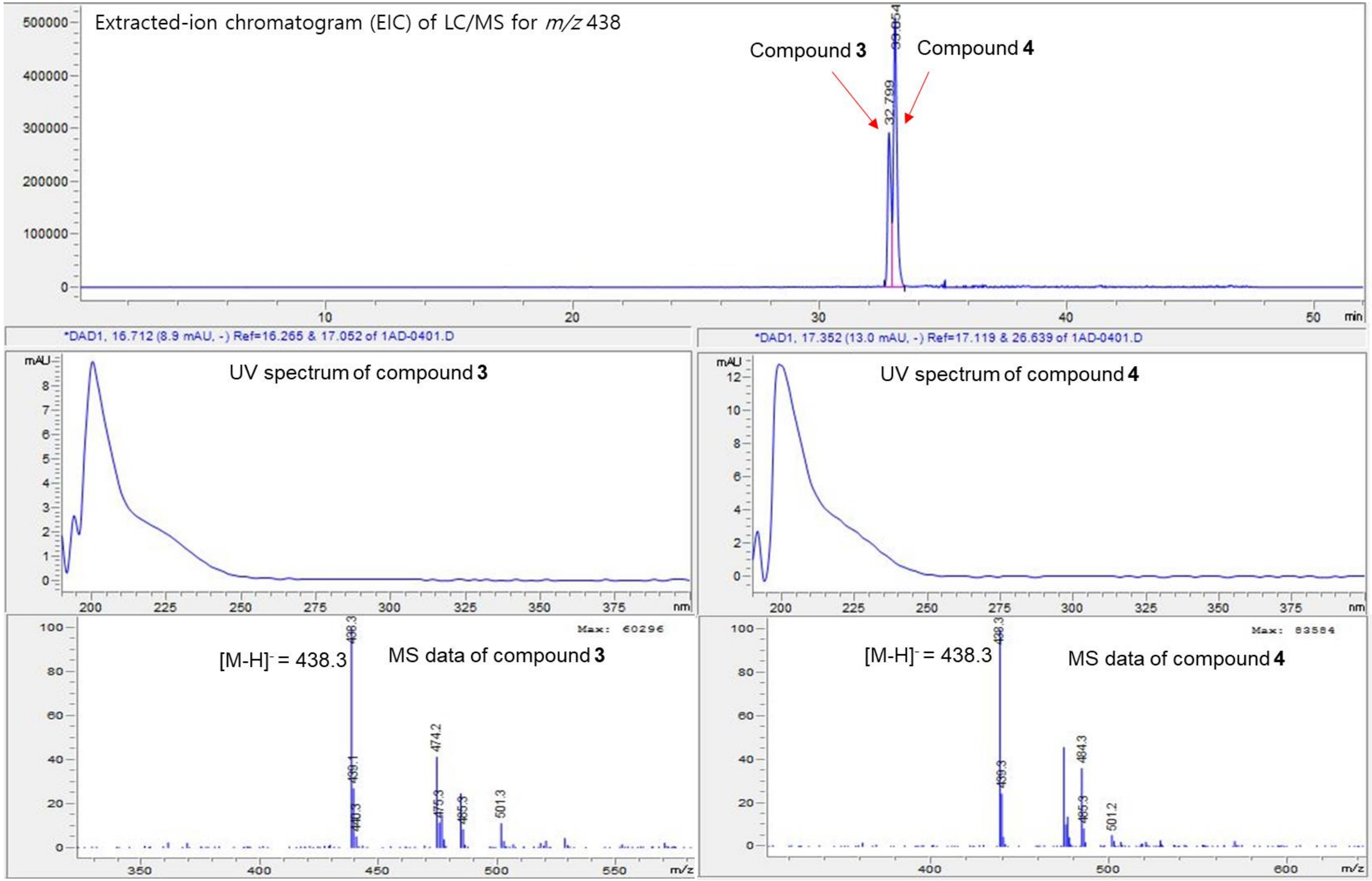

Supplement: Supplementary file 1 [file molecules-23-02568-s001.pdf]
